# Housekeeping gene *gyrA*, a potential molecular marker for *Bacillus* ecology study

**DOI:** 10.1186/s13568-022-01477-9

**Published:** 2022-10-26

**Authors:** Yan Liu, Polonca Štefanič, Youzhi Miao, Yansheng Xue, Weibing Xun, Nan Zhang, Qirong Shen, Ruifu Zhang, Zhihui Xu, Ines Mandic-Mulec

**Affiliations:** 1 Jiangsu Provincial Key Lab of Solid Organic Waste Utilization, Jiangsu Collaborative Innovation Center of Solid Organic Wastes, Educational Ministry Engineering Center of Resource-Saving Fertilizers, The Key Laboratory of Plant Immunity, College of Resources and Environmental Sciences, Nanjing Agricultural University, Nanjing, 210095 People’s Republic of China; 2 Department of Microbiology, Biotechnical Faculty, University of Ljubljana, Ljubljana, Slovenia

**Keywords:** Molecular marker, 16S rRNA, Housekeeping *gyrA* gene, SNPs, *Bacillus* diversity

## Abstract

*Bacillus* is a genus of microorganisms (bacteria) and contains many important commercial species used in industry, agriculture and healthcare. Many different *Bacilli* are relatively well understood at the single-cell level; however, molecular tools that determine the diversity and ecology of *Bacillus* community are limited, which limits our understanding of how the *Bacillus* community works. In the present study, we investigated the potential of the housekeeping gene *gyrA* as a molecular marker for determining the diversity of *Bacillus* species. The amplification efficiency for *Bacillus* species diversity could be greatly improved by primer design. Therefore, we designed a novel primer pair *gyrA*3 that can detect at least 92 *Bacillus* species and related species. For *B. amyloliquefaciens*, *B. pumilus,* and *B. megaterium*, we observed that the high variability of the *gyrA* gene allows for more detailed clustering at the subspecies level that cannot be achieved by the 16S rRNA gene. Since *gyrA* provides better phylogenetic resolution than 16S rRNA and informs on the diversity of the *Bacillus* community, we propose that the *gyrA* gene may have broad application prospects in the study of *Bacillus* ecology.

**Supplementary Information:**

The online version contains supplementary material available at 10.1186/s13568-022-01477-9.

## Introduction

Microbial communities in soil are known to be one of the largest reservoirs of biological diversity and have been extensively studied (Timmis and Ramos 2021). Current advances in high-throughput DNA sequencing of a portion of the small subunit of ribosomal RNA (16S and 18S rRNA) form the backbone of most studies of soil microbial ecology (Klindworth et al. 2013). For bacteria, the 16S rRNA gene is usually preferred, because it contains both highly conserved and hypervariable regions (Peer et al. 1996), and especially because comprehensive reference databases have been compiled for comparison (McDonald et al. 2012; Quast et al. 2013; Cole et al. 2014; Yoon et al. 2017). However, 16S rRNA amplicon sequencing also has many shortcomings: first, 16S rRNA evolves slowly and is highly conserved, making it a poor marker for distinguishing between closely related strains. Second, chimera formation during PCR is high because 16S rRNA variability is very low (Pinto and Raskin 2012; Sun et al. 2013). Third, the number of 16S rRNA copies in different species is highly variable, and single nucleotide polymorphisms (SNPs) at the single-cell level may result in an overestimation of diversity (Johnson et al. 2019). Fourth, the similarity between species can be very high, making it difficult to delineate species in cluster analysis, and different clustering levels lead to different results (Edgar 2013). Therefore, new complementary taxonomic markers for genetic and bioinformatic analysis need to be developed to study microbial diversity in more detail, especially at the subspecies level.

*Bacillus* is one of the most intensely studied bacterial genus comprising at least 200 species (Mandic-Mulec et al. 2015). It is a heterogeneous bacterial taxon that is ubiquitous in various ecological niches and widely used in medicine, industry and agriculture. Although there have been many in-depth studies on *Bacillus* model species, community-level studies on *Bacillus* in soil and other habitats lag. Because sequences of 16S rRNA within *Bacillus* species are often similar, the definition and delineation of bacterial species based on the 16S rRNA comparison among related species in the genus *Bacillus* are unclear. Therefore, the identification and typing of *Bacillus* isolates based on the 16S rRNA gene alone cannot provide accurate results and it is important to explore and use other genes as molecular markers to assess the diversity of the *Bacillus* community (Mandic-Mulec et al. 2015).

Housekeeping genes are potential candidates for assessing microbial diversity because they have been shown to elicit higher phylogenetic resolution than the 16S rRNA gene, such as the *rpoB* gene, *gyrA* gene, and *gyrB* gene, etc. (Chun and Bae 2000; Kasai et al. 2000; Yamamoto et al. 2000; Hurtle et al. 2004; Stefanic and Mandic-Mulec 2009; Stefanic et al. 2012, 2015; Levican et al. 2013; Ménard et al. 2016). Typically, there are only one or two copies of housekeeping genes per genome, and the use of low-copy number genes compared to high-copy number genes could lead to more accurate diversity analysis by avoiding overestimation of diversity due to SNPs in different gene copies. Indeed, several studies have tested the *rpoB* gene and the *gyrB* gene as molecular markers to analyze the diversity of bacterial communities by amplicon sequencing. The results showed that housekeeping gene sequencing provided a more accurate description of bacterial community composition than 16S rRNA sequencing under certain conditions (Vos et al. 2012; Poirier et al. 2018; Ogier et al. 2019).

The housekeeping gene *gyrA*, encoding DNA gyrase subunit A, is essential for DNA replication and is present in all bacteria (Cozzarelli 1980). Analyses of *gyrA* identity provided higher phylogenetic resolution than the 16S rRNA gene for tested *Bacillus* isolates (Chun and Bae 2000; Ménard et al. 2016). Specifically, partial *gyrA* gene sequences were used for phylogenetic analysis and species identification of seven *Bacillus* strains, including *B. amyloliquefaciens*, *B. atrophaeus*, *B. licheniformis*, *B. mojavensis*, *B. subtilis*, *B. subtilis* subsp. *spizizenii,* and *B. vallismortis* (Chun and Bae 2000). Moreover, the *gyrA* gene sequences provided a good marker for *B. subtilis* and *B. amyloliquefaciens* and showed better discriminatory potential between these two closely related species than the *rpoB* gene (Chun and Bae 2000). The *gyrA* gene has been also successfully used to detect intraspecific diversity of *B. subtilis* isolates from soil microscale (Stefanic and Mandic-Mulec 2009) and tomato rhizosphere isolates (Oslizlo et al. 2015). Overall, these works suggest that *gyrA* has a good potential to be used as a molecular marker for microbial ecology studies of the *Bacillus* genus and related species, however, to the best of our knowledge, the available primer pairs used in the studies indicated above, have not been used for amplicon-based community analyses of *Bacillus* species.

In this study, we first compared the rate of variation of the 16S rRNA and *gyrA* genes between 20 *Bacillus* species and then designed a new primer pair that specifically target *gyrA* (*gyrA*3). We then evaluate their performance by using in silico PCR, testing their efficiency on *Bacillus* isolates and performing SNPs analysis of 16S rRNA and *gyrA* genes for selected species that were available in the NCBI database. Finally, verified *gyrA*3 primers to differentiate species and strains of *Bacillus* mock community, and compared the obtained results with those targeting 16S rRNA. Our results suggest that the *gyrA* gene is a useful molecular marker for the identification of *Bacillus* isolates and describing the diversity of the *Bacillus* community.

## Materials and methods

### Strains and culture condition

The 127 strains (56 species) used in the study were strains of *Bacillus* (32 species) and related genera of *Bacillus* (*Paenibacillus, Lysinibacillus, Aneurinibacillus, Virgibacillus, Brevibacillus, Halobacillus* and *Fictibacillus*; 24 species), all strains were isolated from soil (Additional file 2: Table S1). All strains were grown at 30 °C in low-salt Luria–Bertani medium (LB), containing 10 g tryptone, 5 g yeast extract, and 3 g NaCl per litre.

### Nucleotide diversity (Pi) analysis

For interspecies analysis, the whole sequences of the 16S rRNA and the *gyrA* genes of 20 *Bacillus* species (361 genomes) were downloaded from the NCBI genome database (Additional file 2: Table S2). For intraspecies analysis, 3 species were selected and whole sequences of 16S rRNA and *gyrA* gene were downloaded from the database: *B. amyloliquefaciens* (88 genomes), *B. licheniformis* (71 genomes) and *B. pumilus* (97 genomes) (Additional file 2: Table S2). The alignment of these sequences was conducted using the online alignment tool Kalign on EMBL (https://www.ebi.ac.uk/Tools/msa/). Subsequently, nucleotide diversity (Pi) was estimated with DnaSP 6 (v.6) using a window size of 100 bp and a step size of 10 bp (Rozas et al. 2017).

### DNA extraction of strains, PCR and gel electrophoresis

Genomic DNA was extracted using the Omega Bacterial DNA Kit D3350 (Omega, Bio-tek, Norcross, GA, USA), and the concentration and quality of DNA were determined using a NanoDrop 2000 spectrophotometer (Wilmington, DE, USA). The reaction mixture for PCR amplification was prepared in 25 μL containing 1 μL of DNA, 2 μL of each primer (the primer pair *gyrA*1 were *gyrA*1-F: 5′-CAGTCAGGAAATGCGTACGTCCTT-3′ (Roberts et al. 1994) and *gyrA*1-R: 5′- GTATCCGTTGTGCGTCAGAGTAAC-3′ (Ansaldi et al. 2002), the primer pair *gyrA*2 were *gyrA*2-F: 5′-CAGTCAGGAAATGCGTACGTCCTT-3′ (Roberts et al. 1994) and *gyrA*2-R: 5′-CAAGGTAATGCTCCAGGCATTGCT-3′ (Roberts et al. 1994), the primer pair *gyrA*3 were *gyrA*3-F: 5′-GCDGCHGCNATGCGTTAYAC-3′ and *gyrA*3-R: 5′-ACAAGMTCWGCKATTTTTTC-3′, the primers for the 16S rRNA gene were 27F: 5′-AGAGTTTGATCCTGGCTCAG-3ʹ and 1492R: 5′-GGTTACCTTGTTACGACTT-3ʹ), 12.5 μL Green Taq Mix (http://www.vazyme.com), and 7.5 μL deionized water. PCR has performed under the following conditions: Predenaturation at 94 °C for 5 min; denaturation at 94 °C for 30 s; annealing at 50 °C for 30 s; elongation at 72 °C for 40 s (35 cycles); and elongation at 72 °C for 7 min.

### In silico* PCR*

The *gyrA* sequence database contains 5062 full-length *gyrA* gene sequences (226 *Bacillus* species), which were downloaded from the NCBI database using the NCBI-genome-download script (https://github.com/kblin/ncbigenome-download/) (Additional file 2: Table S3). First, the degenerate primer pair *gyrA*3 was converted to primers that do not contain degenerate bases, and the converted *gyrA*3 is listed in Additional file 2: Table S4. Subsequently, the primer pairs *gyrA*1, *gyrA*2 and the converted *gyrA*3 were aligned with the *gyrA* database using NCBI-blast + software (v.2.9.0). The match at 18 bases of both, the forward and reverse primer, was considered amplifiable by the primer pair.

### Phylogenetic analysis

In this study, phylogenetic analysis of genes was performed using MEGA (v.5.05) (Tamura et al. 2011) for Neighbor-Joining and the reliability of clades was tested using 1000 bootstrap replicates. Furthermore, annotation and beautification of trees were performed using programs available at the iTol online site (https://itol.embl.de) (Letunic and Bork 2019).

### SNPs analysis

A total of 600 available genomes of 4 different *Bacillus* species were obtained using the same method as in-silico PCR, including 116 genomes of *B. amyloliquefaciens*, 140 genomes of *B. pumilus*, 117 genomes of *B. megaterium* and 226 genomes of *B. anthracis* (Additional file 2: Table S5). The alignment of the 16S rRNA (base sites: 330–810) and *gyrA* genes (base sites: 350–850) within each species was performed using the L-INS-I method of MAFFT (v7.487) (https://mafft.cbrc.jp/alignment/software/) (Katoh et al. 2002). The 2 gene sequences (16S rRNA, *gyrA*) on the same genome were selected as representative sequences, and the base mismatch sites on other sequences were marked with color after comparison with the representative sequence.

### Construction of the DNA-based Bacillus mock community, amplicon sequencing and data analysis

For the *Bacillus* mock community, we selected 8 strains with known genome sequences. Genomic DNA from 8 strains was extracted and its quality and quantity were determined. Eight genomic DNAs were pooled in equal amounts after being diluted to approximately the same concentrations. The hypervariable region V3-V4 of the 16S rRNA gene was amplified with the universal primers 338F: 5′-CCTACGGRRBGCASCAGKVRVGAAT-3’ and 806R: 5′-GGACTACNVGGGTWTCTAATCC-3′. The *gyrA* gene from 8 strains of the mock community was amplified with primer pairs *gyrA*3 (see above), *gyrA*4 (F: 5′-TAYGCRATGAGYRTHATYGT-3’ and R: 5′- TTBGTNGCCATHCCDACMGC-3ʹ), and *gyrA*5 (F: 5′-GCDGCNGCVATGCGTTAYAC-3ʹ and R: 5′- CGNAGRTYBGTAATDCCDTC-3ʹ). Sequencing was performed on an Illumina Miseq PE300 instrument.

Raw data were processed using the Unoise3 algorithm (Edgar 2016) in the UPARSE pipeline (http://drive5.com/usearch/manual/uparse_pipeline.html) (Edgar 2013) to obtain the ZOTUs represent sequences and the ZOTUs table. The ZOTUs represent sequences were annotated using the sequences of 16S rRNA and *gyrA* gene of the eight strains (Additional file 2: Dataset S1, S2). The ZOTUs of the same strain were pooled into a single unit after annotation. Finally, we used box plots to show the community structure and the characteristics of the different primer pairs during sequencing. The box plots were drawn using R (v.4.0.3) (R Core Team 2020).

## Results

### The gyrA gene of the Bacillus genus shows higher variation rates than 16S rRNA

**Fig. 1:**
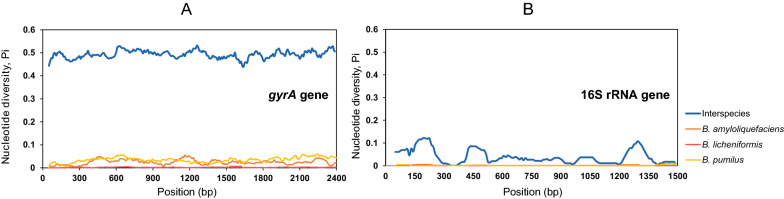
Sliding window analysis of nucleotide variability [Pi (π)] along the sequence of the *gyrA* gene (**A)** and 16S rRNA (**B)** in *Bacillus*. 361 and 256 of *gyrA* and 16S sequences from 20 species and three species (*B. amyloliquefaciens* (n = 88), *B. licheniformis* (n = 71) and *B. pumilus* (n = 97)) were aligned with the Kalign method and nucleotide diversity was determined in DnaSP (v.6) using a window size of 100-bp, a step size of 10-bp, and points based on the mid-point of each window (i.e., the first point is at position 200). The blue lines display the *Bacillus* inter-species nucleotide diversity of the two genes and the orange, red and yellow lines display the nucleotide diversity of two genes in *B. amyloliquefaciens*, *B. licheniformis and B. pumilus,* respectively. The sequence length of the *gyrA* gene for analysis was 2450 bp, and that of 16S rRNA was 1540 bp

The housekeeping gene *gyrA* is considered to be more variable than 16S rRNA and has been used as a molecular tool for the classification and identification of *B. subtilis* species (Chun and Bae 2000; Borshchevskaya et al. 2013). In the genus *Bacillus*, the nucleotide diversity (Pi) of 16S rRNA and the *gyrA* gene sequences were 0.039 and 0.491, respectively (Fig. 1A, B blue line). It indicated that the degree of interspecies variation was significantly higher for the *gyrA* gene than for the 16S rRNA gene.

In three *Bacillus* species, the intraspecific nucleotide diversity (Pi) of 16S rRNA was again significantly lower than the intraspecific nucleotide diversity of *gyrA* gene sequences in all three species: *B. amyloliquefaciens* (Pi_16S_ = 0.0014; Pi_*gyrA*_ = 0.0244), *B. licheniformis* (Pi_16S_ = 0.00024; Pi_*gyrA*_ = 0.0021) and *B. pumilus* (Pi_16S_ = 0.00136; Pi_*gyrA*_ = 0.0344) (Fig. 1A, B nonblue lines).

In conclusion, the *Bacillus gyrA* gene shows higher variation rates than 16S rRNA, hence we propose that *gyrA* represents a promising molecular marker for analyses of *Bacillus* community diversity analyses and the diversity of *Bacillus* isolates.

### First comparative tests of three primer pairs for the detection of Bacillus species

**Fig. 2:**
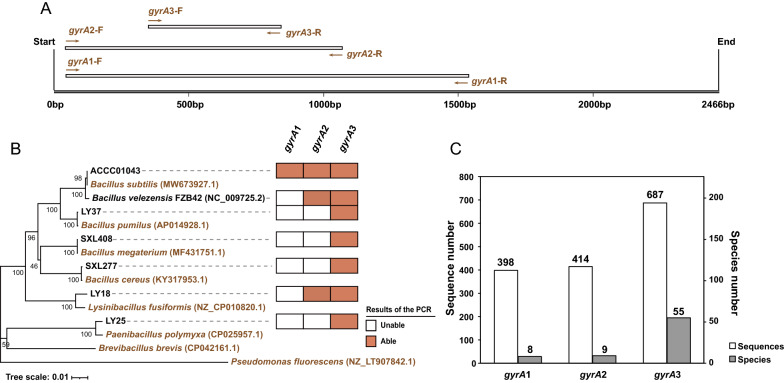
Displays the ability of the three primer pairs to amplify the *gyrA* gene for seven *Bacillus* strains. **A** The position of *gyrA* amplicons was obtained by primer pairs *gyrA*1, *gyrA*2 and *gyrA*3. **B** PCR amplification of seven *Bacillus* strains using three *gyrA* primer pairs. The orange square indicates that the target gene of *gyrA* was amplified by the indicated primer pair, and the white square indicates that it was not. The phylogenetic tree was constructed based on the complete 16S rRNA (1358 bp) (Additional file 2: Dataset S3) by using the Neighbor-Joining method in MEGA 5.05 software. The reliability of clades was tested by the 1000 bootstrap replications. **C** Display of the results of the computer simulation amplification ability of the three *gyrA* primer pairs using the *gyrA* sequence database. The white columns represent the number of sequences in the database that were matched by the primers, while the grey columns represent the number of *Bacillus* species identified by the primer pairs (Additional file 2: Table S6). The *gyrA* sequences in database were downloaded from the NCBI genome database (Additional file 2: Table S3)

As indicated above *Bacillus* isolates have been already analyzed by primers targeting *gyrA*, however the specificity of these primers has not been investigated broadly (Roberts et al. 1994; Ansaldi et al. 2002). To satisfy the amplicon sequencing requirements, we designed a new primer pair (*gyrA*3) (Fig. 2A), and compared its amplification potential in colony PCR and virtual PCR with the previously designed primers, referred to here as *gyrA*1 and *gyrA*2 (Fig. 2A) (Roberts et al. 1994; Ansaldi et al. 2002).

First, we selected seven strains of different *Bacillus* species: *L. fusiformis*, *P. polymyxa*, *B. pumilus*, *B. velezensis*, *B. megaterium*, *B. cereus* and *B. subtilis* (Fig. 2B) to perform PCR amplification with primer pairs *gyrA*1, *gyrA*2 and *gyrA*3. The PCR amplification results showed that *gyrA*1 detected only *B. subtilis*; *gyrA*2 detected *B. subtilis*, *B. velezensis* and *L. fusiformis*; whereas *gyrA*3 performed much better and detected all *Bacillus* species included in the analysis (Fig. 2B and Additional file 1: Fig. S1).

The in-silico PCR analysis was performed using the *gyrA* gene database containing 226 *Bacillus* species. The results showed that only 8 *Bacillus* species were amplified in-silico by *gyrA*1, 9 *Bacillus* species were amplified by *gyrA*2 (Fig. 2C and Additional file 1: Fig. S2), while 55 *Bacillus* species were amplified by *gyrA*3 (Fig. 2C). The majority of sequences amplified by *gyrA*1 and *gyrA*2 belonged to *B. subtilis*, whereas *gyrA*3 demonstrated broader diversity as evidenced by the amplification of seven species and in-silico PCR (Fig. 2B, C and Additional file 2: Table S6).

### *Specificity range of the gyrA3 primer pair by using PCR and *in silico* PCR*

**Fig. 3:**
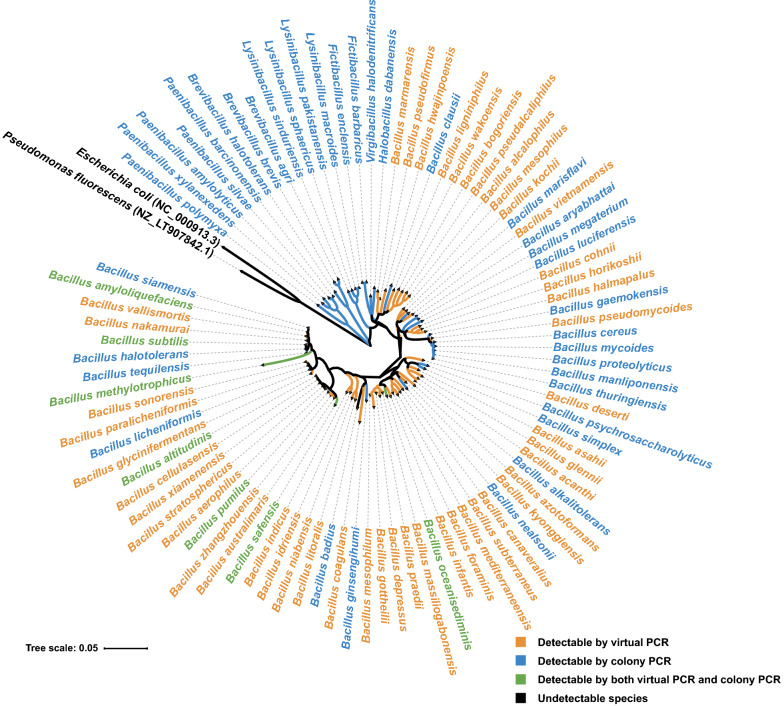
The amplification ability of the primer pair *gyrA*3 of *Bacillus* species and related species performed by computer simulation and in situ amplification. The *Bacillus* species indicated by the orange color (48 species) represent the species in the database that were matched by *gyrA*3 only in virtual PCR. The *Bacillus* species in blue color represented strains that can be amplified by *gyrA*3 only by colony PCR. These strains included 21 *Bacillus* species and 16 *Bacillus*’s related genera species. The *Bacillus* species in green color (7 species) represent strains that can be amplified by two methods: virtual PCR and colony PCR. The species in black color (2 species) represent strains that cannot be amplified in virtual PCR nor by colony PCR. The phylogenetic tree was constructed based on 16S rRNA (1302 bp)

Because the *gyrA*3 primer pair performed better than the previously reported primers, we next combined analysis of the in-silico amplified *gyrA* genes with PCR analysis of *Bacillus* isolates from our laboratory culture collection. Virtual *gyrA*3 PCR amplicons from 55 different *Bacillus* species from the *gyrA* gene database were evenly distributed among the branches of the phylogenetic tree (Fig. 3 orange and green).

Next, we used 127 strains of *Bacillus* (32 species) and related genera (*Paenibacillus*, *Lysinibacillus*, *Aneurinibacillus*, *Virgibacillus*, *Brevibacillus*, *Halobacillus*, *Fictibacillus*, 24 species) from our laboratory culture collection to amplify their *gyrA* genes with a *gyrA*3 primer pair (Additional file 2: Table S1). The results showed that 28 *Bacillus* species and 16 *Bacillus*-related species could be amplified by the *gyrA*3 primers (Fig. 3 blue and green), while the remaining 4 *Bacillus* species and 8 *Bacillus*-related species could not be amplified by the *gyrA*3 primers. Of these *Bacillus* species, marked in green in the phylogenetic tree, 7 species were detectable by both methods (Fig. 3 green). In summary, the primer pair *gyrA*3 can potentially detect 76 *Bacillus* species and as many as 16 species from related genera (Fig. 3).

### The gyrA gene provides better intraspecific phylogenetic resolution than the 16S rRNA gene among certain species

Compared to 16S rRNA, the molecular evolution rate of *gyrA* gene sequences is faster (Timmis and Ramos 2021), so we hypothesized that *gyrA* might provide better phylogenetic resolution at the subspecies level. The single nucleotide polymorphisms (SNPs) analysis of the 16S rRNA (V3-V4 region) and the *gyrA* gene (*gyrA*3 amplicon region) was performed in four *Bacillus* species (*B. amyloliquefaciens*, *B. pumilus*, *B. megaterium* and *B. anthracis*). We did not include an analysis of the *B. subtilis* genomes because templates for *gyrA* primers have already been developed and applied for analyses of this species (Roberts et al. 1994; Ansaldi et al. 2002; De Clerck et al. 2004; Stefanic and Mandic-Mulec 2009).

**Fig. 4:**
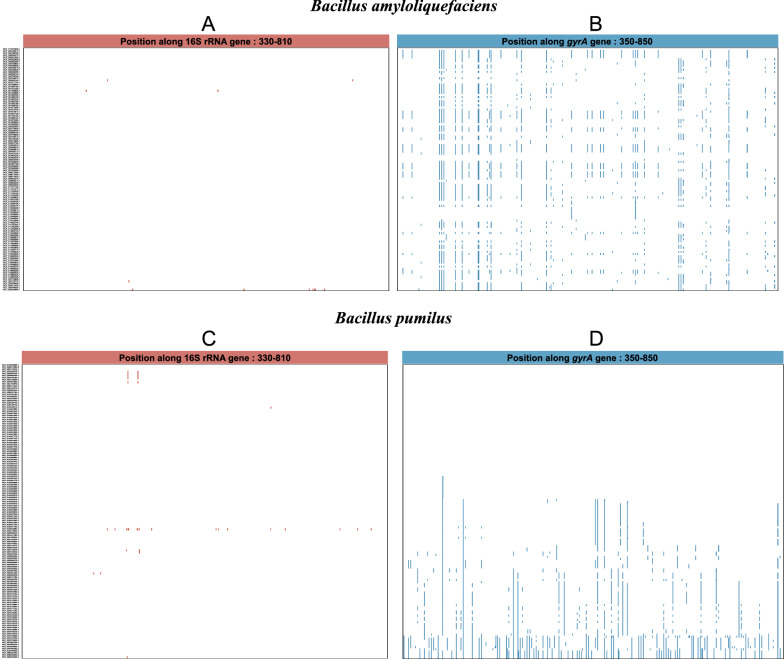
Alignment of the 16S rRNA and *gyrA* sequences of *B. amyloliquefaciens* (116 genomes) and *B. pumilus* (140 genomes). Sequences were aligned using L-INS-I method of MAFFT (v7.487). The analysis involved positions along 16S rRNA from 330–810 and along *gyrA* from 350–850. On the left side of the graph GCF reference numbers of genomes were displayed, with reference sequence GCF_017815555.1 displayed at the top for *B. amyloliquefaciens* and GCF_014269965.1 for *B. pumilus*. The display of base site variation was drawn using MEGA (v.5.05). The colored line (red or blue) indicates a variation of specific base sites as compared to the reference sequence

In *B. amyloliquefaciens*, the 480 bp long 16S rRNA region (V3-V4 region) contained 12 variable base sites (Fig. 4A and Additional file 1: Fig. S3A), which were detected in only 4 of 116 genomes of this species (Fig. 4A), and the SNPs frequency at variable sites within the 4 genomes ranged from 0.21%-1.46% (Additional file 1: Fig. S3C red column). In contrast, the 500-nucleotide *gyrA* region (positions 350–850) contained 59 variable sites (Fig. 4B and Additional file 1: Fig. S3B). The variable sites were detected in 109 of 116 genomes (Fig. 4B), and the frequency of SNPs at the variable sites ranged from 0.4% to 6.4% (Additional file 1: Fig. S3C blue column).

In *B. pumilus*, alignment of the 16S rRNA V3-V4 region revealed 21 variable base sites (Fig. 4C and Additional file 1: Fig. S4A), but again only in 11 out of 140 genomes (Fig. 4C). The frequency of SNPs at variable sites ranged from 0.21% to 3.54% (Additional file 1: Fig. S4C red column). In contrast, in the *gyrA* gene, 130 of the base sites were variable (Fig. 4D and Additional file 1: Fig. S4B) and these were found in 87 of 140 genomes, with SNPs frequencies at variable sites ranging from 0.2% to 15.8% (Additional file 1: Fig. S4C blue column). However, in *B*. *pumilus* nearly 50% of the genomes examined had 100% identity in the *gyrA* gene, indicating a high degree of relatedness between genomes that may require sequencing of additional marker genes for clonality verification and strain typing.

We also observed that the variation of the *gyrA* gene is quite different in different *Bacillus* species, which could be a bias of the NCBI database or property of certain species. For example, *B. megaterium* showed lower diversity with 3 bases of variation in the 16S rRNA alignment region (V3-V4 region) (Additional file 1: Fig. S5A and Fig. S6A). Although 48 of 117 genomes showed polymorphism, the maximum SNPs frequency at variable sites of *B. megaterium* genomes was only 0.42% (Additional file 1: Fig. S6C red column). The *gyrA* gene was again more polymorphic with 58 bases of variation (Additional file 1: Fig. S5B, S6B) occurring in 99 of 117 genomes, with SNPs’ frequencies at variable sites ranging from 0.2% to 2.8% (Additional file 1: Fig. S6C blue column).

The variation divergence between the 2 genes of *B. anthracis* was much lower than in the three *Bacillus* species described above. Although we identified 13 variable base sites in the 16S rRNA V3-V4 region and 65 variable base sites in the *gyrA* gene region (Additional file 1: Fig. S5C, D and Additional file 1: Fig. S7A, B), the SNPs occurred in only 7 and 18 of 226 genomes, respectively. Moreover, the SNPs frequencies at variable sites in 7 and 18 of *B. anthracis* genomes were also low: 0.21%-1.04% and 0.2%-7.4%, respectively (Additional file 1: Fig. S7C).

Overall, our results showed that within *Bacillus* species the frequency of SNPs in the *gyrA* gene was consistently much higher than in the 16S rRNA (Fig. 4 and Additional file 1: Fig. S5). We therefore suggest that the *gyrA* gene provides better resolution than 16S rRNA for identification and typing of *Bacillus* isolates at the subspecies level. This is particularly true for *B. amyloliquefaciens*, *B. pumilus* and *B. megaterium* but less so for *B. anthracis* (Table 1).

**Table 1:** Polymorphisms of the 16S rRNA and *gyrA* gene

Species	Gene	Gene length (bp)	Number of variable sites	Total number of genomes	Genomes number with variable bases	Range of genomes variable sites	Range of genomes variable sites (%)
*B. amyloliquefaciens*	16S	480	12	116	4	1–7	0.21–1.46
	*gyrA*	500	59		109	2–32	0.4–6.4
*B. pumilus*	16S	480	21	140	11	1–17	0.21–3.54
	*gyrA*	500	130		87	1–79	0.2–15.8
*B. megaterium*	16S	480	3	117	48	1–2	0.21–0.42
	*gyrA*	500	58		99	1–14	0.2–2.8
*B. anthracis*	16S	480	13	226	7	1–5	0.21–1.04
	*gyrA*	500	65		18	1–37	0.2–7.4

### The resolution power of Bacillus mock community gyrA amplicon sequencing

**Fig. 5:**
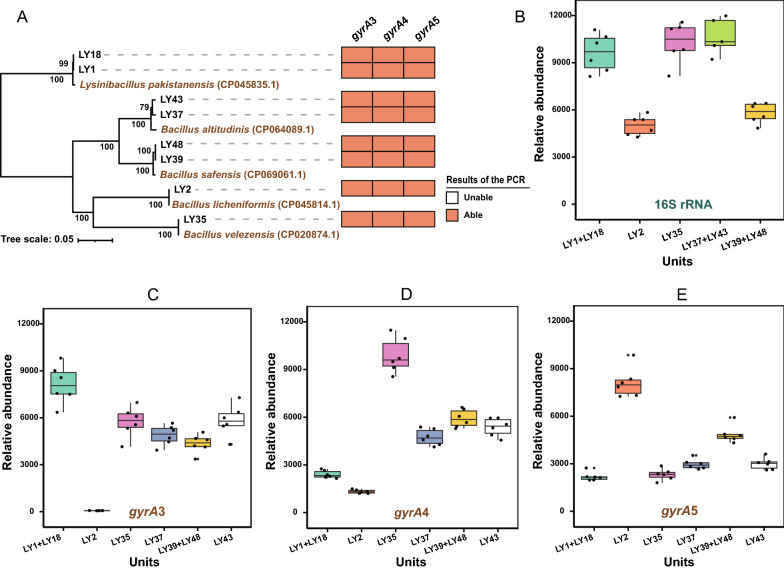
The results of amplicon sequencing for the mock community including eight *Bacillus* strains. **A** PCR amplification of eight *Bacillus* strains by *gyrA* gene primer pairs. The orange square indicates positive and white square unsuccessful amplification. The amplicon sequencing results of 16S rRNA gene **B** and *gyrA* gene using primer pairs *gyrA*3 **C**, *gyrA*4 **D**, and *gyrA*5 **E** for the mock community. The relative abundance is represented by reads numbers for each unit as indicated on the X axis. The phylogenetic tree was contracted based on the complete *gyrA* genes (2469 bp) (Additional file 2: Dataset S2) by using the Neighbor-Joining method in MEGA 5.05 software. The reliability of clades was tested by the 1000 bootstrap replications. The box plots were drawn by the ggplot2 R package (v.3.2.1)

Our results above suggest that the amplicon sequence of the primer pair *gyrA*3 could be used as a molecular marker for diversity analysis of *Bacillus*. Next, we aimed to design a mock community to test the efficacy of the primers *gyrA*3 and used the general 16S rRNA primers (V3-V4) as a positive control. For better comparison, we designed 2 additional primer pairs, *gyrA*4 and *gyrA*5, which are very close to the position of the *gyrA*3 in the *gyrA* gene (Additional file 1: Fig. S8). We selected eight strains belonging to four species (*B. altitudinis*, *B. licheniformis*, *B. velezensis* and *L. pakistanensis*) and successfully amplified their *gyrA* gene by *gyrA*3, *gyrA*4 and *gyrA*5 primer pairs in a routine PCR for selected strains (Fig. 5A).

Next, we constructed a mock community of eight strains to retest the resolution power of the three *gyrA* primer pairs and the 16S rRNA-specific primers. Our goal was to test whether the primer pair is suitable for determining the diversity of the mock community (Fig. 5). Sequencing of the 16S rRNA amplicons showed that 16S rRNA primers could distinguish only five units, as strains LY1 and LY18, LY37 and LY43, and LY39 and LY48 had identical V3-V4 nucleotide sequence (Fig. 5B). The *gyrA* primer pairs were capable of resolving 6 units, but *gyrA*4 and *gyrA*5 produced amplicons of variable abundance and preferentially amplified LY35 and LY2, respectively (Fig. 5C–E). In comparison, primers *gyrA*3 also amplified six fragments but the relative abundance of these amplicons was more uniform (Additional file 2: Table S7) with the exception of LY2 strain (Fig. 5C). Our data suggest that *gyrA*3 has potential for Illumina amplicon sequencing of more complex *Bacillus* communities.

## Discussion

It is believed that the diversity of microorganisms in nature is immense, so its detection remains a challenge (Widder et al. 2016). Molecular tools (e.g., for specific amplification of marker genes) combined with high-throughput sequencing are expected to open the door to the vast diversity of microorganisms (Klindworth et al. 2013). Here, we systematically investigated the potential of the *gyrA* gene as a marker gene for the taxonomic typing of *Bacillus* isolates and assessment of *Bacillus* community composition by amplicon sequencing. The novel *gyrA*3 primer pair is capable of detecting 76 *Bacillus* species by virtual and colony PCR; hence, our results suggest that the *gyrA* gene is a good phylogenetic marker for detecting intra- and interspecific diversity of the genus *Bacillus*.

Although 16S rRNA is widely used as a molecular marker for bacterial community analyses (Clarridge 2004), its amplicon sequencing can only describe community diversity at the genus level (Gupta et al. 2019). This is particularly true for *Bacillus* species, which exhibit very low interspecific variability (Vos et al. 2012). In contrast, faster evolution and consequently higher diversity of the *gyrA* gene (Timmis and Ramos 2021) suggests that this gene might provide higher phylogenetic resolution than the 16S rRNA gene within the genus *Bacillus*. In our study, the differential effect of the *gyrA* gene on *Bacillus* interspecies and several *Bacillus* species was better than that of 16S rRNA (Fig. 1). Moreover, the results of comparative sequence analysis (16S rRNA V3-V4 region and the *gyrA*3 amplicon region of the *gyrA* gene) of the four species showed the wider range of SNPs in the *gyrA* genes than in the 16S rRNA (Fig. 4, Additional file 1: Fig. S5 and Table 1). Our results are consistent with findings that housekeeping genes, including *gyrA*, evolve much faster than 16S rRNA genes and are suitable for the identification and typing of closely related species (Poirier et al. 2018) and the intraspecific resolution of isolates, as previously shown for *B. subtilis* (Roberts et al. 1994; Ansaldi et al. 2002). Because protein-coding genes involved in DNA processing have evolved differently than rRNA, protein translation is affected by the degenerative codes, and nucleotide changes may propagate along genes without affecting amino acid sequence. Therefore, housekeeping genes encoding proteins are more powerful than 16S rRNA in distinguishing between highly related strains (Navarro and Martínez-Murcia 2018).

To date, there have been only a few reports in which conserved genes (e.g., *rpoB* and *gyrB*) have been used as templates for amplicon sequencing of microbial communities (Vos et al. 2012; Poirier et al. 2018; Ogier et al. 2019). These reports show that sequencing of conserved protein-coding genes provides a more accurate description of bacterial community composition than 16S rRNA sequencing. Specifically, the *rpoB* gene has been used in addition to the 16S rRNA molecular marker for high-throughput sequencing studies of species diversity in the *Proteobacteria* phylum (Vos et al. 2012).

Caution should be recommended when using single protein-coding genes as molecular markers, as they may exhibit different phylogenetic resolutions or may be subjected to possible horizontal gene transfer or recombination processes (Navarro and Martínez-Murcia 2018). However, many previous studies have reported the application of the *gyrA* gene in the identification and typing of *Bacillus* strains, so the *gyrA* gene should not have the above-mentioned concerns (Chun and Bae 2000; Hurtle et al. 2004; Stefanic and Mandic-Mulec 2009; Stefanic et al. 2012, 2015). And we identified here its resolution power for 76 *Bacillus* species (Fig. 3) and propose that the diversity of the *Bacillus* community can be more accurately assessed by combining 16S rRNA and *gyrA* amplicon sequencing.

As a molecular marker, the housekeeping gene *gyrA* also has some limitations, unlike ribosomal RNA, due to the high variability of protein-encoding housekeeping gene sequences, the design of universal sequencing primers is not always achievable (Schleifer 2009). Therefore, the inability of primer pair *gyrA*3 to amplify strains of the entire *Bacillus* genus is also expected, as has been previously described for the *Escherichia* genus (Johnning et al. 2015). Besides, the choice of the amplified region (ie, the design of the primers) affects the discrimination of the *Bacillus* species by the *gyrA* gene. The *gyrA* amplicon region we selected was well suited for resolving subspecies of *B. amyloliquefaciens*, *B. pumilus*, and *B. megaterium*. For *B. anthracis,* which is known for its low diversity (Lista et al. 2006) intraspecific resolution was limited (Fig. 4 and Additional file 1: Fig. S5). Moreover, in the high-throughput amplicon sequencing of eight strains, the primer pair *gyrA*3 also showed different advantages from the primer pairs *gyrA*4 and *gyrA*5 (Fig. 5). The primer pair *gyrA*3 also had its blind area in the detection, this could be due to the extremely high similarity of the *gyrA* genes in selected genomes, some of which have even identical *gyrA* sequences. However, the *gyrA*3 distinguished very well two strains with highly similar *gyrA* genes such as LY37 and LY43, which 16S rRNA did not (Fig. 5). Although primers for amplicon sequencing of the *gyrA* gene had certain limitations, such as not amplifying all selected targets or not reflecting the abundance of added DNA, this study has put forward the advantages that *gyrA*3 covers the broadest diversity of *Bacillus* species reported to date.

In summary, this study investigated the application of the *gyrA* gene as a molecular marker in *Bacillus* subspecies typing and high-throughput sequencing of the *Bacillus* mock community. The greater ability of *gyrA*-based analyses to distinguish *Bacillus* strains at the subspecies level should increase resolution and provide more reliable results for the ecological studies of the genus *Bacillus*. We believe that the primer pair will have broad applications in *Bacillus* research.

## Supplementary Information

**Additional file 1: Figure S1** The agarose gel electrophoresis of DNA amplification products of three *gyrA* gene primer pairs: *gyrA*1 **(A)**, *gyrA*2 **(B)** and *gyrA*3 **(C)**. 1-7 indicates strains LY18, LY25, LY37, FZB42, SXL408, SXL277 and ACCC01043. **Figure S2 **The amplification ability of the primer pair* gyrA*1 and *gyrA*2 in *Bacillus* species by computer simulation. The eight *Bacillus* species were amplified by *gyrA*1 and nine *Bacillus* species were amplified by *gyrA*2. The phylogenetic trees were constructed based on *gyrA* gene (2403 bp). **Figure S3 **Polymorphisms in the 16S rRNA and *gyrA* gene’s regions of *B. amyloliquefaciens *(116 genomes). **(A)** The proportion of variation at different base sites along the 16S rRNA V3-V4 region. **(B)** The proportion of variation at the different base positions of the *gyrA* gene region. **(C) **The proportion of variants along 16S rRNA (red column) and *gyrA* gene (blue column) region in different genomes. **Figure S4 **Polymorphisms in the 16S rRNA and *gyrA* gene’s region of *B.* *pumilus* (140 genomes). **(A)** The proportion of variation at different base sites along the 16S rRNA **(B)** and the *gyrA* gene. **(C) **The proportion of variable base sites in 16S rRNA (red column) and *gyrA* (blue column) sequences in different genomes. **Figure S5 **Alignment of the 16S rRNA and *gyrA* sequences of *B.* *megaterium* (117 genomes) and *B.* *anthracis* (226 genomes). Sequences were aligned using L-INS-I method of MAFFT (v7.487). The analysis involved positions along 16S rRNA from 330-810 and along *gyrA* from 350-850. On the left side of the graph GCF reference numbers of genomes were displayed, with reference sequence GCF_002577645.1 displayed at the top for *B.* *megaterium* and GCF_000007845.1 for *B. anthracis*. The display of base site variation was drawn using MEGA (v.5.05). The colored line (red or blue) indicates a variation of specific base sites as compared to the reference sequence. **Figure S6 **Polymorphisms in the 16S rRNA and *gyrA* gene’s regions of *B.* *megaterium* (117 genomes). **(A)** The proportion of variation at different base sites along the 16S rRNA V3-V4 region. **(B)** The proportion of variation at the different base positions of the *gyrA* gene region. **(C) **The proportion of variants along 16S rRNA (red column) and *gyrA* gene (blue column) region in different genomes. **Figure S7** Polymorphisms in the 16S rRNA and *gyrA* gene’s region of *B.* *anthracis* (226 genomes). **(A)** The proportion of variation at different base sites along the 16S rRNA gene **(B)** and the *gyrA* gene. **(C) **The proportion of variable base sites in 16S rRNA (red column) and *gyrA* (blue column) sequences in different genomes. **Figure S8 **Description of *gyrA* gene primer pairs for amplicon sequencing. (**A)** The position of *gyrA* amplicons was obtained by primer pairs *gyrA*3, *gyrA*4 and *gyrA*5. (**B)** PCR amplification of eight *Bacillus* strains by *gyrA* gene primer pairs. The orange square indicates positive and white square unsuccessful amplification. The phylogenetic tree was contracted based on the complete *gyrA* genes (2469 bp) (Supplemental Dataset S2) by using the Neighbor-Joining method in MEGA 5.05 software. The reliability of clades was tested by the 1000 bootstrap replications.

**Additional file 2:** **TableS1** The information of 127 strains for colony PCR. **TableS2** The genomes information for nucleotide variability analysis of *gyrA* gene and 16S rRNA gene in inter- and intraspecies *Bacillus*. **TableS3** The *gyrA* gene database information of *Bacillus* for computer simulation. **TableS4** The general primers converted from degenerate primer pair *gyrA3*. **TableS5** The genomes information for SNP analysis of *gyrA* gene and 16S rRNA gene in four *Bacillus* species. **TableS6** The information of sequences amplified by three *gyrA* gene primer pairs in computer simulation. **TableS7** The analysis results of mock community amplicon sequencing. **DatasetS1** The 16S rRNA gene sequences of the eight *Bacillus* strains for mock *Bacillus* community. **DatasetS2** The *gyrA* gene sequences of the eight *Bacillus* strains for mock *Bacillus* community. **DatasetS3** The 16S rRNA gene sequences of the seven *Bacillus* strains.

## Data Availability

Data and materials used in the analysis are available upon request from the corresponding authors for the purposes of reproducing or extending the analysis. Amplicon sequencing reads from the 16S rRNA gene and *gyrA* gene are available at NCBI Sequence Read Archive under accession number PRJNA823863.
