# Supplementary material for: Housekeeping gene gyrA, a potential molecular marker for Bacillus ecology study
Source: AMB Express. 2022 Oct 26;12:133. doi: 10.1186/s13568-022-01477-9 (PMC9606167; doi:10.1186/s13568-022-01477-9)
Supplement: Supplementary file 1 — Additional file 1: Figure S1 The agarose gel electrophoresis of DNA amplification products of three gyrA gene primer pairs: gyrA1 (A), gyrA2 (B) and gyrA3 (C). 1-7 indicates strains LY18, LY25, LY37, FZB42, SXL408, SXL277 and ACCC01043. Figure S2 The amplification ability of the primer pair gyrA1 and gyrA2 in Bacillus species by computer simulation. The eight Bacillus species were amplified by gyrA1 and nine Bacillus species were amplified by gyrA2. The phylogenetic trees were constructed based on gyrA gene (2403 bp). Figure S3 Polymorphisms in the 16S rRNA and gyrA gene’s regions of B. amyloliquefaciens (116 genomes). (A) The proportion of variation at different base sites along the 16S rRNA V3-V4 region. (B) The proportion of variation at the different base positions of the gyrA gene region. (C) The proportion of variants along 16S rRNA (red column) and gyrA gene (blue column) region in different genomes. Figure S4 Polymorphisms in the 16S rRNA and gyrA gene’s region of B. pumilus (140 genomes). (A) The proportion of variation at different base sites along the 16S rRNA (B) and the gyrA gene. (C) The proportion of variable base sites in 16S rRNA (red column) and gyrA (blue column) sequences in different genomes. Figure S5 Alignment of the 16S rRNA and gyrA sequences of B. megaterium (117 genomes) and B. anthracis (226 genomes). Sequences were aligned using L-INS-I method of MAFFT (v7.487). The analysis involved positions along 16S rRNA from 330-810 and along gyrA from 350-850. On the left side of the graph GCF reference numbers of genomes were displayed, with reference sequence GCF_002577645.1 displayed at the top for B. megaterium and GCF_000007845.1 for B. anthracis. The display of base site variation was drawn using MEGA (v.5.05). The colored line (red or blue) indicates a variation of specific base sites as compared to the reference sequence. Figure S6 Polymorphisms in the 16S rRNA and gyrA gene’s regions of B. megaterium (117 genomes). (A) The proportion of va [file 13568_2022_1477_MOESM1_ESM.docx]

Title: Housekeeping gene *gyrA*, a potential molecular marker for *Bacillus* ecology study

Journal name: AMB Express

Yan Liu^1^, Polonca Štefanič^2^, Youzhi Miao^1^, Yansheng Xue^1^, Weibing Xun^1^, Nan Zhang^1^, Qirong Shen^1^, Ruifu Zhang^1^, Zhihui Xu^1*^, Ines Mandic-Mulec^2^

1 Jiangsu Provincial Key Lab of Solid Organic Waste Utilization, Jiangsu Collaborative Innovation Center of Solid Organic Wastes, Educational Ministry Engineering Center of Resource-Saving Fertilizers, The Key Laboratory of Plant Immunity, Nanjing Agricultural University, 210095 Nanjing, Jiangsu, Peoples R China

2 Department of Microbiology, Biotechnical Faculty, University of Ljubljana, Ljubljana, Slovenia

*Corresponding authors: Zhihui Xu

College of Resources and Environmental Sciences, Nanjing Agricultural University, Nanjing, 210095, P.R. China.

E-mail: xzh2068@njau.edu.cn, Tel: 86-025-84396177, Fax: 86-025-84396260


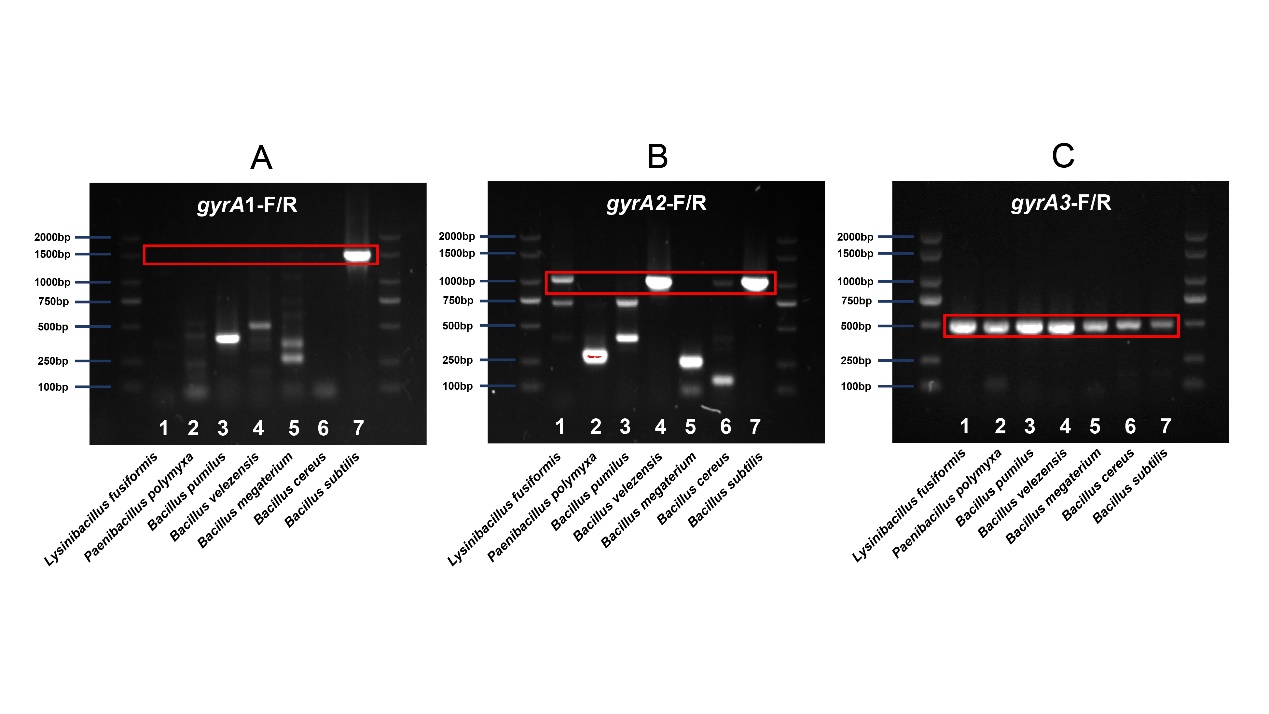


**Figure S1** The agarose gel electrophoresis of DNA amplification products of three *gyrA* gene primer pairs: *gyrA*1 **(A)**, *gyrA*2 **(B)** and *gyrA*3 **(C)**. 1-7 indicates strains LY18, LY25, LY37, FZB42, SXL408, SXL277 and ACCC01043.


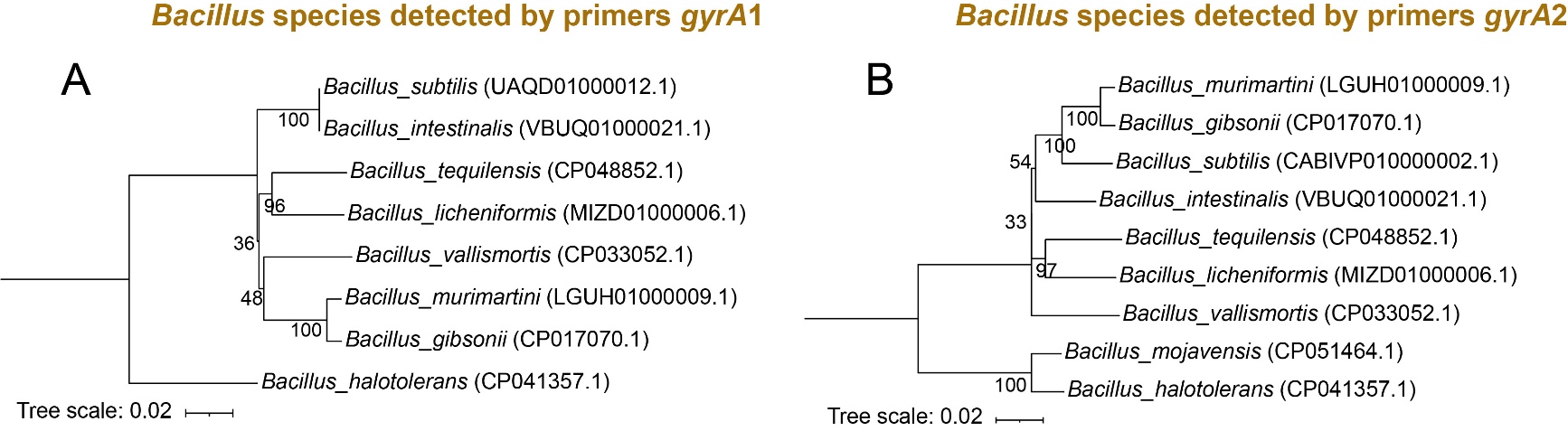


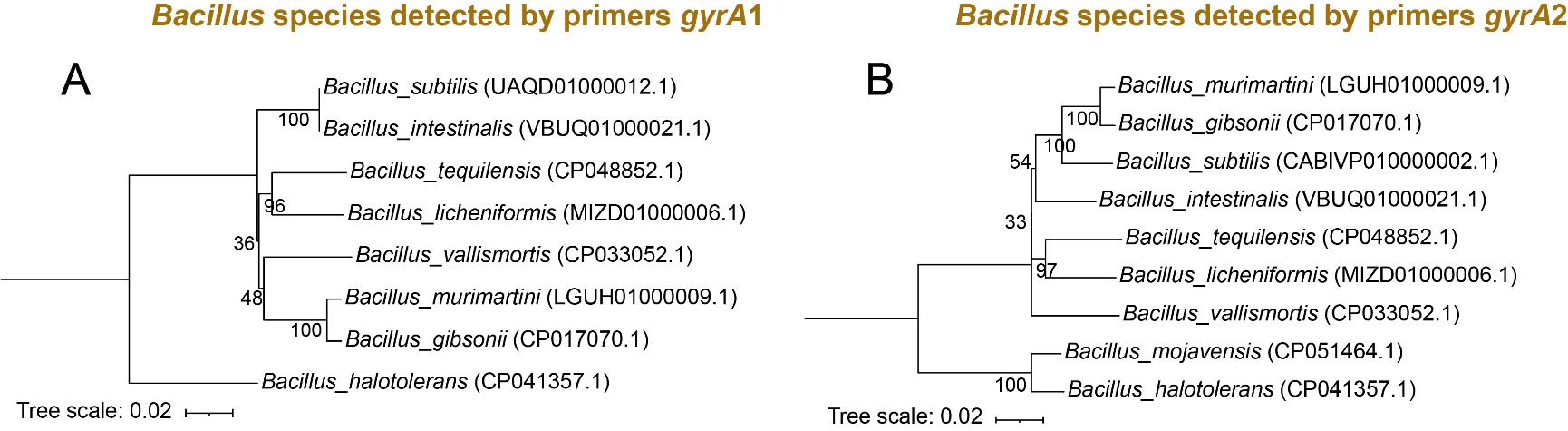


**Figure S2** The amplification ability of the primer pair *gyrA*1 and *gyrA*2 in *Bacillus* species by computer simulation. The eight *Bacillus* species were amplified by *gyrA*1 and nine *Bacillus* species were amplified by *gyrA*2. The phylogenetic trees were constructed based on *gyrA* gene (2403 bp).


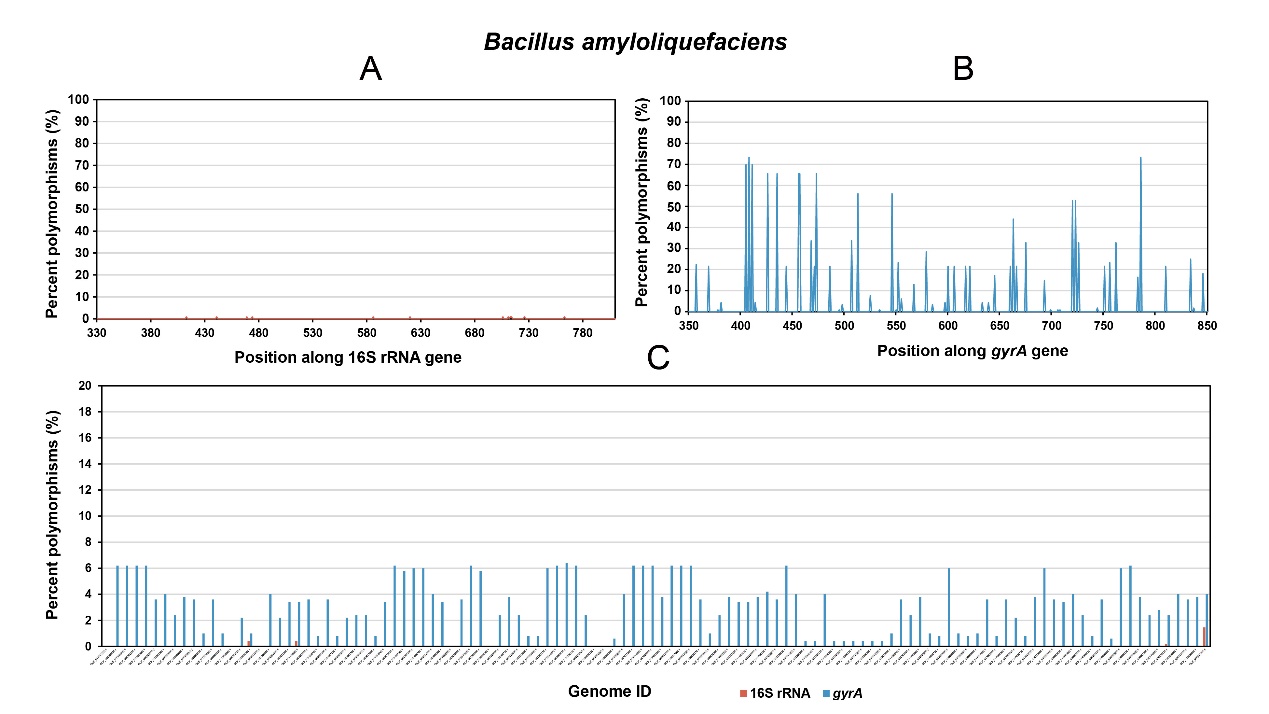


**Figure S3** Polymorphisms in the 16S rRNA and *gyrA* gene’s regions of *B. amyloliquefaciens* (116 genomes). **(A)** The proportion of variation at different base sites along the 16S rRNA V3-V4 region. **(B)** The proportion of variation at the different base positions of the *gyrA* gene region. **(C)** The proportion of variants along 16S rRNA (red column) and *gyrA* gene (blue column) region in different genomes.


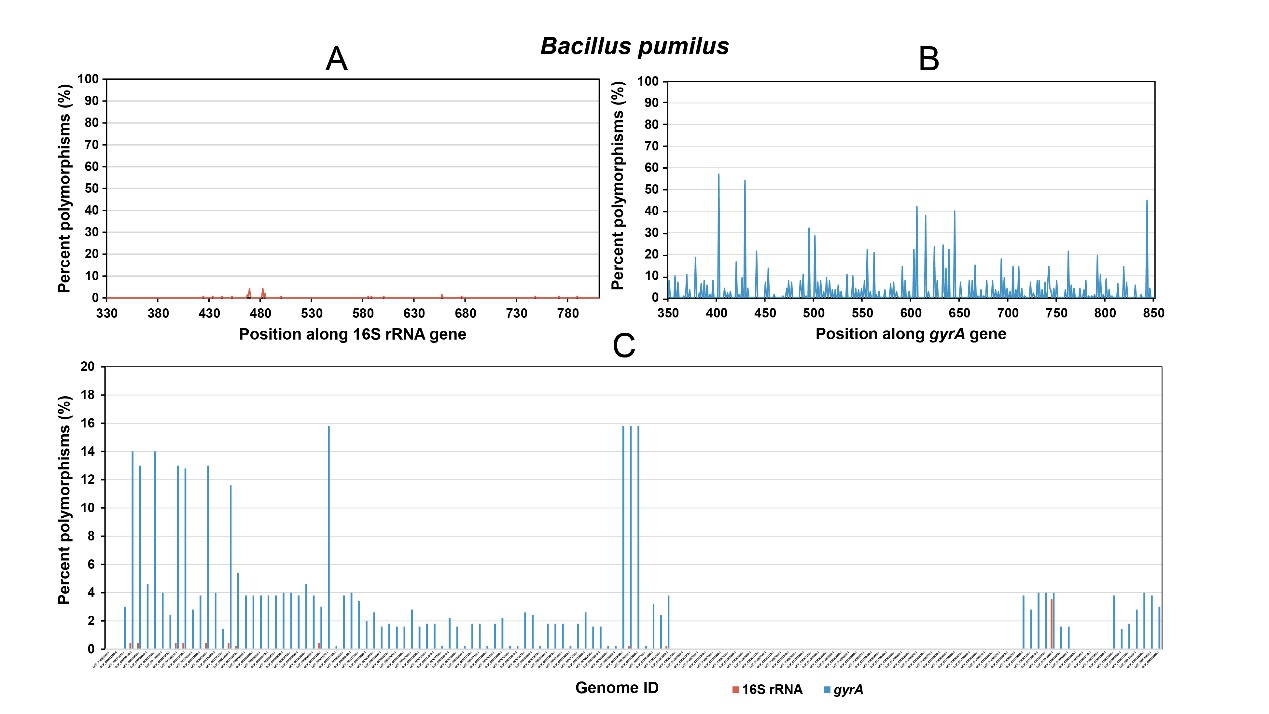


**Figure S4** Polymorphisms in the 16S rRNA and *gyrA* gene’s region of *B. pumilus* (140 genomes). **(A)** The proportion of variation at different base sites along the 16S rRNA **(B)** and the *gyrA* gene. **(C)** The proportion of variable base sites in 16S rRNA (red column) and *gyrA* (blue column) sequences in different genomes.


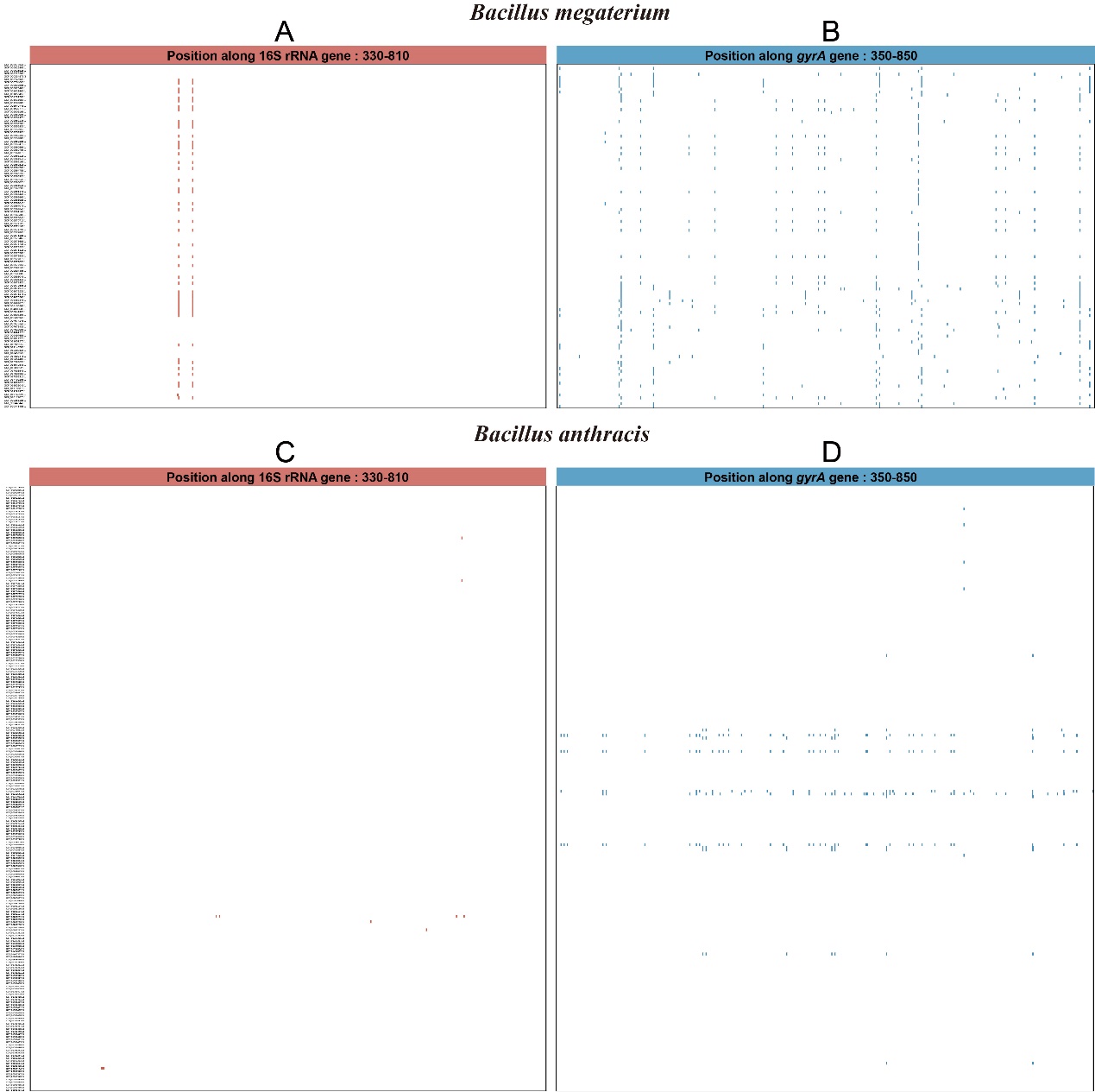


**Figure S5** Alignment of the 16S rRNA and *gyrA* sequences of *B.* *megaterium* (117 genomes) and *B. anthracis* (226 genomes). Sequences were aligned using L-INS-I method of MAFFT (v7.487). The analysis involved positions along 16S rRNA from 330-810 and along *gyrA* from 350-850. On the left side of the graph GCF reference numbers of genomes were displayed, with reference sequence GCF_002577645.1 displayed at the top for *B.* *megaterium* and GCF_000007845.1 for *B. anthracis*. The display of base site variation was drawn using MEGA (v.5.05). The colored line (red or blue) indicates a variation of specific base sites as compared to the reference sequence.


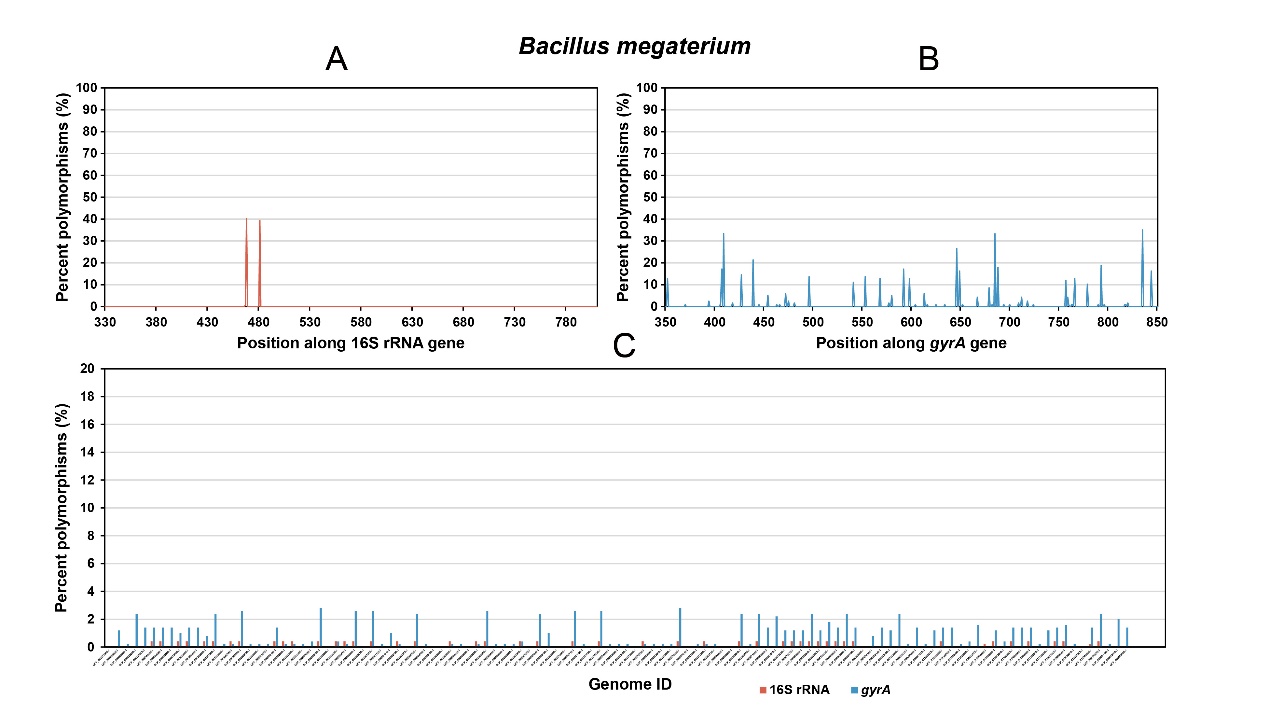


**Figure S6** Polymorphisms in the 16S rRNA and *gyrA* gene’s regions of *B.* *megaterium* (117 genomes). **(A)** The proportion of variation at different base sites along the 16S rRNA V3-V4 region. **(B)** The proportion of variation at the different base positions of the *gyrA* gene region. **(C)** The proportion of variants along 16S rRNA (red column) and *gyrA* gene (blue column) region in different genomes.


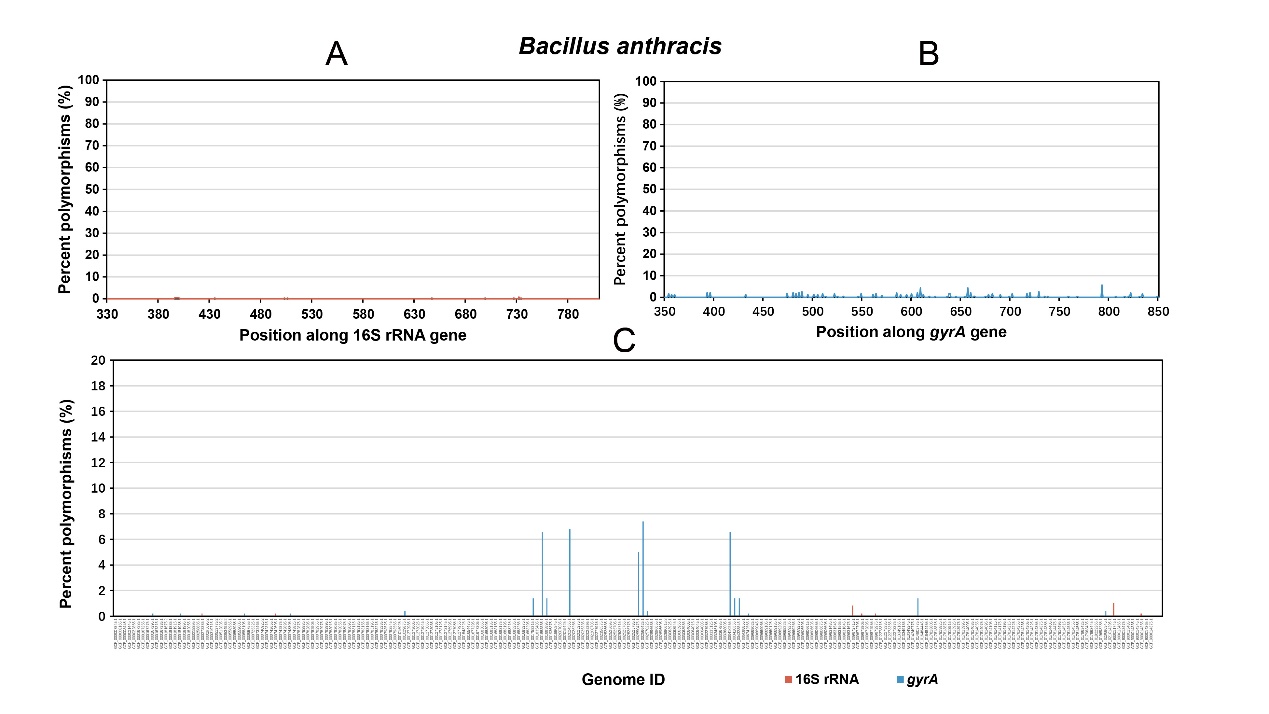


**Figure S7** Polymorphisms in the 16S rRNA and *gyrA* gene’s region of *B. anthracis* (226 genomes). **(A)** The proportion of variation at different base sites along the 16S rRNA gene **(B)** and the *gyrA* gene. **(C)** The proportion of variable base sites in 16S rRNA (red column) and *gyrA* (blue column) sequences in different genomes.


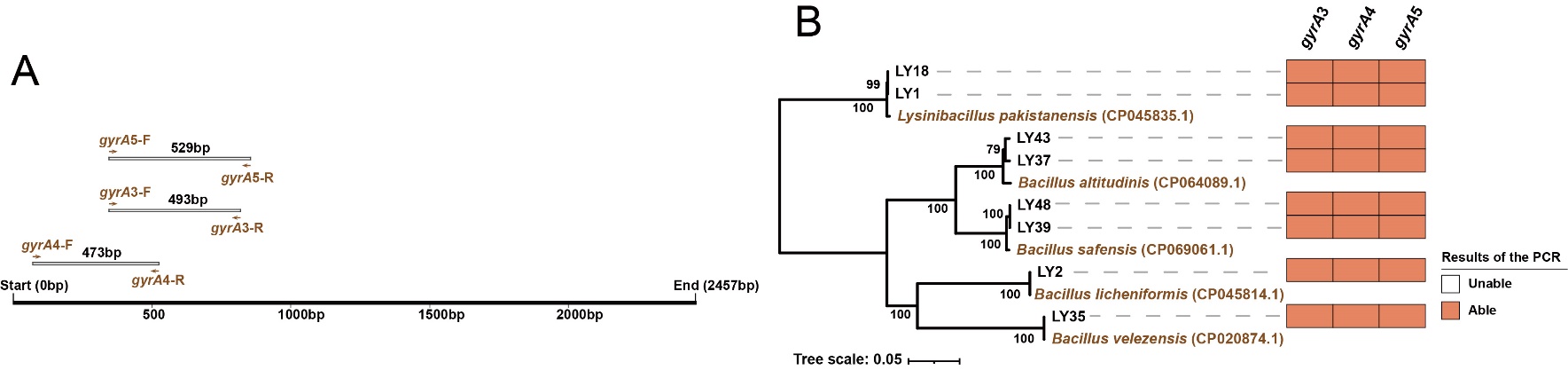


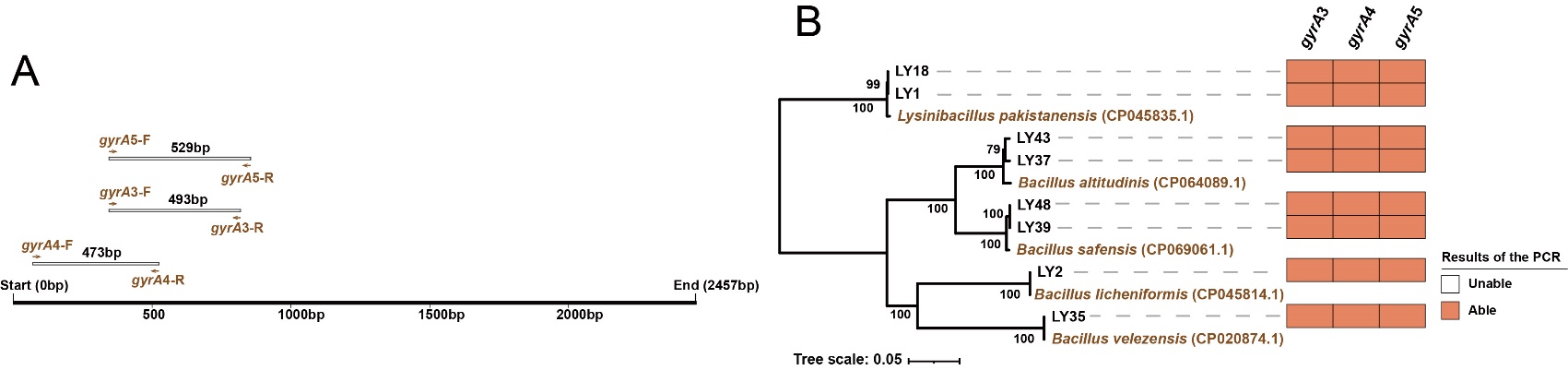


**Figure S8** Description of *gyrA* gene primer pairs for amplicon sequencing. (**A)** The position of *gyrA* amplicons was obtained by primer pairs *gyrA*3, *gyrA*4 and *gyrA*5. (**B)** PCR amplification of eight *Bacillus* strains by *gyrA* gene primer pairs. The orange square indicates positive and white square unsuccessful amplification. The phylogenetic tree was contracted based on the complete *gyrA* genes (2469 bp) (Supplemental Dataset S2) by using the Neighbor-Joining method in MEGA 5.05 software. The reliability of clades was tested by the 1000 bootstrap replications.
